# LAG-3 expression in tumor microenvironment of triple-negative breast cancer

**DOI:** 10.55730/1300-0144.5567

**Published:** 2022-11-20

**Authors:** Gözde TAHTACI, Nazan GÜNEL, Aysu SADİOĞLU, Nalan AKYÜREK, Oğulcan BOZ, Aytuğ ÜNER

**Affiliations:** 1 Department of Medical Oncology, Faculty of Medicine, Gazi University, Ankara, Turkey; 2 Department of Pathology, Faculty of Medicine, Gazi University, Ankara, Turkey; 3 Department of Internal Medicine, Faculty of Medicine, Gazi University, Ankara, Turkey

**Keywords:** Breast cancer, LAG-3, PD-L1, tumor infiltrating lymphocytes, triple-negative

## Abstract

**Background/aim:**

This study aimed to evaluate the expression of lymphocyte activation gene-3 (LAG-3) and its relationship with programmed cell death ligand-1 (PD-L1) in triple-negative breast cancer (TNBC).

**Materials and methods:**

LAG-3 and PD-L1 was evaluated in tumor-infiltrating lymphocytes (TILs) using immunohistochemistry (IHC). The chi-square test was used to estimate the associations between LAG-3, PD-L1 and clinicopathological characteristics. Correlation between LAG-3 stromal TIL (sTIL), LAG-3 intraepitelial TIL (iTIL) and PD-L1 was assessed with using the Spearman’s correlation coefficient. Survival analysis was performed using the Kaplan-Meier method.

**Results:**

The percentages of LAG-3 sTIL+, LAG-3 iTIL+, PD-L1+ tumor cells and PD-L1+ inflammatory cells were 52%, 42%, 14% and 70%, respectively. A strong positive correlation between LAG-3 sTIL and LAG-3 iTIL (r = 0.874, p < 0.001) and a moderate positive correlation between LAG-3 sTIL and PD-L1 (r = 0.584, p < 0.001) were found. LAG-3 and PD-L1 status did not significantly affect overall survival (OS) (HR: 0.56 (95% CI: 0.15–2.11) (p = 0.397), HR: 2.70 (95% CI: 0.56–13.02) (p = 0.215), respectively).

**Conclusion:**

High levels of LAG-3 and PD-L1 expression were detected in patients with TNBC. Although their contribution to survival could not be determined, the high expression rates of PD-L1 and LAG-3 may help identify the subgroup of TNBC that would benefit from immunotherapy.

## 1. Introduction

Breast cancer is the most common type of cancer in women worldwide [1]. According to GLOBOCAN data, while 2.3 million new cases of breast cancer were diagnosed in 2020; it is estimated to reach 3.19 million by 2040 [2]. Although there has been a decrease in breast cancer mortality as a result of cancer screening programs and new treatments, mortality rate of triple-negative breast cancer (TNBC) remains high. TNBC, which constitutes 10%–20% of breast cancers, is associated with a worse prognosis among all breast cancer subtypes [3]. The aggressive behavior of tumor, lack of biomarkers, and druggable targets also contribute to a poor prognosis.

Evaluation of the tumor microenviroment (TME), which plays an important role in the development and progression of cancer, reveals new targets for cancer treatment. Breast cancer is considered a nonimmunogenic tumor; however, recent studies have shown that TNBC is highly immunogenic [4]. TILs are important members of the TME and consist of CD4 + T cells, CD8 + T cells, T regulatory cells, B cells, natural killer (NK) cells, dendritic cells, and macrophages. In TNBC, the presence of TILs has been found to be associated with a better response to chemotherapy and a longer survival [5,6]. However, the prognostic effect of TILs in immunotherapeutic agent treatment remains unclear.

Programmed cell death-1 (PD-1) is a transmembrane protein expressed in T, B, and NK cells. It regulates the balance between inhibitory and stimulatory signals and maintains effective immunity and self-tolerance [7]. By binding to one of its ligands, programmed cell death ligand-1 (PD-L1) increases T-cell apoptosis and dysfunction and decreases the cellular immune response [8]. There are conflicting results in the literature regarding the prognostic value of PD-L1 in breast cancer [4, 9,10].

Lymphocyte activation gene 3 (LAG-3), an inhibitory protein discovered in 1990, is expressed in T cells, B cells, NK cells, dendritic cells and TILs, and plays a role in autoimmune diseases, chronic infections and cancer [11]. It prevents T cell activation and reduces the development of antitumor responses by binding to major histocompatibility complex (MHC) class II [12]. Several studies have reported LAG-3 expression in TNBC [13–15]. However, as a result of different pathological methods and scoring systems for LAG-3 assessment, LAG-3 expression and its effect on survival have not yet been elucidated. In this study, we investigated the expression of LAG-3 and its relationship with PD-L1 in TNBC.

## 2. Materials and methods

### 2.1. Patients

Forty-nine patients diagnosed with nonmetastatic TNBC in our medical oncology clinic between 2008 and 2018 were included. TNBC was defined as estrogen and progesterone receptor negativity and the absence of Her-2 receptor expression by immunohistochemistry or in situ hybridization analysis. Demographic and clinicopathologic data were recorded retrospectively using patient files and hospital information system. Tumor diameter was defined as the largest tumor diameter indicated in the postoperative pathology report. The largest tumor size was recorded in patients who received neoadjuvant therapy according to clinical and imaging studies. Overall survival (OS) was calculated as the time from diagnosis to death, for patients still alive as the time until March 2021. This retrospective study was approved by the Institutional Ethics Committee of Gazi University Medical Faculty (Date: 28.05. 2018, number: 403).

### 2.2. Immunohistochemical analysis

Pretreatment formalin fixed, paraffin embedded tissues were examined for LAG-3, TILs, and PD-L1 expression by immunohistochemistry.

#### 2.2.1. LAG-3 and TILs

From the formalin fixed, paraffin embedded tissues, 4 μm thick sections were obtained with a positively charged slide. The sections were deparaffinized with xylene and rehydrated with ethanol series (100%, 95%, 70%). After these procedures, the sections were prepared for LAG-3 antibodies in order to prepare the tissue for antibody binding in the Ventana Benchmark ULTRA automated immunohistochemistry stainer ethylenediamine tetraacetic acid (EDTA) buffer (pH: 8.0) for 76 min and then LAG-3 (1: 200, D2G40, rabbit monoclonal Cell Signaling Technology, Beverly, MA, USA) antibodies were incubated in the tissues for 1 h for primary antibody incubation. An ultraView Universal 3,3′-diaminobenzidine (DAB) detection kit was used to provide color rendering. In immunohistochemical staining of LAG-3 expression, five large magnification fields for each biopsy were evaluated. Lymphocytes not directly associated with the breast cancer cluster were evaluated as sTIL and lymphocytes within the carcinoma cluster were evaluated as iTIL. Patients with ≥1% of TILs expressing LAG-3 were considered positive.

#### 2.2.2. PD-L1

**Figure 1:**
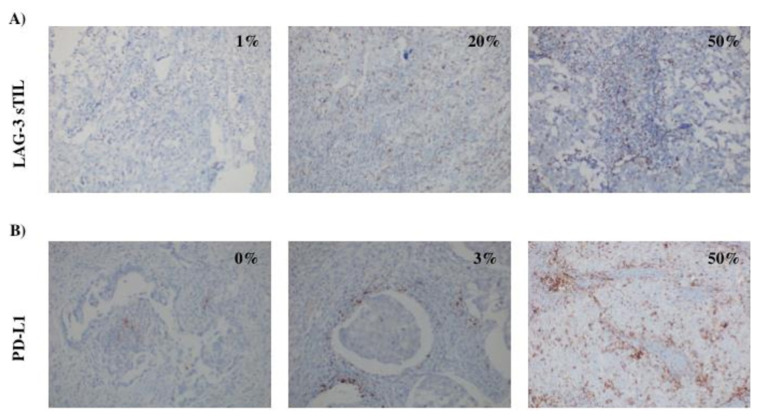
(A) Representative images showing the expressions of LAG-3 sTIL, (B) representative images showing the expressions of PD-L1 in inflammatory cells.

For the PD-L1 antibody (1: 200, SP142, Roche, Tucson, Arizona, USA), sections were kept in EDTA buffer (pH: 8.0) for 64 min and incubated with PD-L1 antibody for 32 min. OptiView detection kit was used. Counterstain with Ventana brand hematoxylin I, washed the slides in tap water, kept in alcohol for 2 min and xylol for 2 min, and then closed using entellan. The stains of tumor cells and inflammatory cells for the PD-L1 staining are given as percentages separately. The cutoff value for PD-L1 positivity was set at 1%. Representative images of PD-L1 and LAG-3 immunohistochemical staining are shown in Figures 1A and 1B.

### 2.3. Statistical analysis

Histogram and Shapiro-Wilks tests were used to analyze the distribution of variables. Frequency, mean ± standard deviation, and median (range) values were calculated according to variables distribution characteristics. The chi-square test and Mann-Whitney U test were used to compare categorical variables between groups. Survival curves were obtained using the Kaplan-Meier method and compared using the log-rank test. Cox proportional hazards model was used to evaluate the effect of variables on survival. Hazard ratios (HRs) estimated using Cox analysis are reported as relative risks with corresponding 95% confidence intervals (CIs). Spearman’s correlation coefficient was used to assess correlation between LAG-3 sTIL, LAG-3 sTIL and PD-L1. p-value of <0.05 was considered statistically significant. Statistical analyses were performed using SPSS version 21.0 (IBM Corporation, Armonk, NY, USA).

## 3. Results

Forty-nine women diagnosed with TNBC were included in this study. The mean age at diagnosis was 52.77 ± 13.21 (26–84) years. Of the patients, 65.3% were postmenopausal women. The mean tumor diameter was 34.20 (4.0–95.0) mm. Angiolymphatic invasion was present in 61.2% of the cases. The most common histological type was invasive ductal carcinoma. Adjuvant chemotherapy, neoadjuvant chemotherapy and adjuvant radiotherapy were applied 73.5%, 22.4% and 74.4% of patients, respectively. The percentages of LAG-3+ sTILs, LAG-3 + iTILs, PD-L1+ immune cells (IC) and PD-L1+ tumor cells (TC) were 55.1%, 40.8%, 69.4% and 14.3%, respectively. Concurrent expression of LAG-3 and PD-L1 was detected in 46.9% of the cases. PD-L1 ICs were preferred for analysis because PD-L1 expression was low in TCs.

The expression of LAG-3 iTILs, LAG-3 sTILs, PD-L1 and their associations with clinicopathological parameters are given in Tables 1 and Table 2. A strong positive correlation between LAG-3 sTIL and LAG-3 iTIL (r = 0.874, p < 0.001) and a moderate positive correlation between LAG-3 sTIL and PD-L1 (r = 0.584, p < 0.001) were found (Table 3).

**Figure 2:**
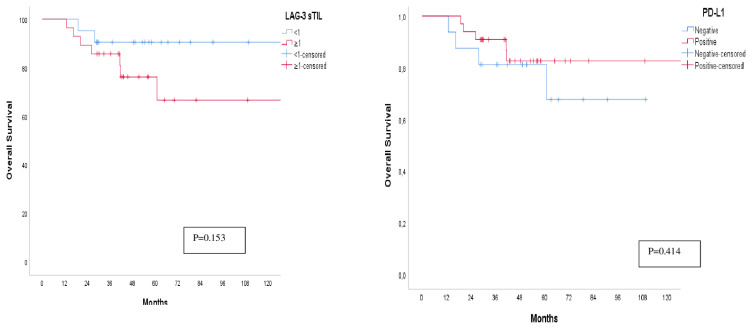
Kaplan-Meier curve of overall survival of (a) LAG-3 sTIL positive and negative patients and (b) PD-L1 positive and negative patients.

The median follow-up time was 47.80 months (range 13.01–130.37 months). There were 10 (10/49) deaths and 13 (10/49) recurrences in this study. Mean overall survival was 120.21 ± 8.17 months (%95 CI: 104.18–136.24). The cumulative survival rate of the entire cohort at 60 months was 76% ± 8%, whereas the cumulative survival rate of LAG-3 iTIL+, LAG-3 iTIL−, LAG-3 sTIL+, LAG-3 sTIL−, PD-L1+ and PD-L1 − patients 50% ± 19%, 89% ± 6%, 62% ± 14%, 90% ± 6%, 83 ± 7%, and 66% ± 15%, respectively. The median OS has not yet been determined. PD-L1 positive and LAG-3 negative cases showed trends towards better prognosis that did not reach statistical significance (HR: 0.58 (95%CI:0.15–2.17) (p = 0.419), HR: 2.94 (95%CI: 0.62–13.96) (p = 0.173), respectively) (Figure 2).

## 4. Discussion

Evaluation of the TME has led to exciting advances in oncology. Immune checkpoints, which play important roles in the TME, are the most important structures targeted in cancer treatment. The use of PD-1/PD-L1 inhibitors, which are the first targeted checkpoints in TNBC, has shown a survival advantage; however, the fact that no response was obtained in most cases led to the idea of targeting different checkpoint inhibitors together or in combination with chemotherapy [16, 17] In this study, we found a high rate of LAG-3 and PD-L1 expressions in TNBC. A strong correlation between LAG-3 sTIL and LAG-3 iTIL expression, and a moderately significant correlation between LAG-3 sTIL and PD-L1 levels was observed. Despite their high expression levels, the effects on survival could not be determined.

LAG-3 plays a crucial role in optimal T-cell regulation. It was introduced approximately two decades ago as a molecule that can be targeted in breast cancer [18]. In preclinical studies, it has been found that there is a synergy between LAG-3 and PD-1; thus, antitumor immunity can be achieved with dual blockage in cases with positivity for both structures [19]. Although there are studies evaluating LAG-3 in many types of cancer, very few studies have evaluated LAG-3 in TNBC. In the present study, LAG-3 and PD-L1 expression levels were higher than those reported in previous studies. There is no definite cutoff value for LAG-3 positivity in the literature. In a study by Burugu et al., the cutoff value was determined to be 1% and LAG-3 iTIL positivity was 33% [13]. However, in the study by Bottai et al., the cutoff value was 5%, and LAG-3 positivity was 18% [14]. In another study, the cutoff value was determined as 20% [20]. This indicates that the assessment of LAG-3 is still experimental, and further studies are needed to determine a precise cutoff value and detection methods. In addition, the heterogeneity of the patients included in the studies could have led to different results. Both sTIL and iTIL have been evaluated in previously. We observed a strong correlation between stromal and intraepithelial LAG-3 expression. Consequently, both of these can be used to assess LAG-3 expression. There was a moderate correlation between LAG-3 sTIL and PD-L1 levels. Indeed, concurrent expression of LAG-3 and PD-L1 was 46.9%; however, the clinical significance of this high ratio has not been clarified.

Another unanswered question is whether LAG-3 expression contributes to survival. However, there are conflicting data in the literature regarding the relationship between LAG-3 expression and survival [13,14, 21]. LAG-3 is expressed in several types of TILs. In preclinical studies, different functions were observed in T cells, NK cells and dendritic cells depending on the TILs in which LAG-3 is expressed [22]. A recent study reported that breast cancer-specific survival is longer in patients with LAG-3+ CD8+ iTILs. Accordingly, the positivity of LAG-3, where it is expressed, is more important [13]. Although high expression levels were detected in this study, their contribution to survival was not observed. This can be explained by the fact that breast cancer is a heterogeneous group, and the different clinical courses can be seen according to the subtypes of TNBC. Therefore, the determination of subgroups of TNBC, molecular and transcriptomic evaluations, and BRCA mutation status will clearly determine the effect of LAG-3 positivity on survival.

LAG-3 inhibitors are currently being tested in clinical trials. Response rates were not sufficient when used as monotherapy for different cancer types [23]. Many clinical trials are ongoing when they are used in combination with chemotherapy or other checkpoint inhibitors, based on the idea that the response rates will increase with combined use with other agents. In line with this idea, a statistically significant increase was found in the pathological complete response rate and event-free survival in the phase 3 KEYNOTE-522 study, in which pembrolizumab was added to neoadjuvant chemotherapy. Furthermore, the benefit of pembrolizumab is independent of PD-L1 expression [24]. In addition to the expression of PD-L1 or LAG-3, changes in the immunoregulatory genes and the transcriptomic profile of the tumor will reveal which patients can benefit more from immunotherapy. TNBC molecular classification should also be considered to clearly evaluate the prognostic and predictive properties of LAG-3 and PD-L1. Molecular heterogeneity and the presence of different oncogenic changes in breast cancer subtypes can cause differences in prognosis and survival [25, 26].

Limitations of this study are its retrospective design, small sample size, and relatively short follow-up period. However, high expression of LAG-3 and PD-L1 suggests that treatment with double blockade would be beneficial.

Although high LAG-3 and PD-L1 expression levels were detected in our study, we could not determine their effects on survival. This suggests that many different mechanisms, beyond the presence of the aforementioned molecules in TME, play a role in breast cancer immunology. Many preclinical and clinical studies are underway for this purpose. We believe that the high expression rates of PD-L1 and LAG-3 may contribute to identifying individuals who would benefit from dual immunotherapy.

## Figures and Tables

**Table 1:** Relationship between LAG-3 iTILs, LAG-3 sTILs, PD-L1 and clinicopathological parameters.

	iLAG-3 − (n = 28)	iLAG-3 + (n = 21)	p	sLAG-3 − (n = 21)	sLAG-3+ (n = 28)	p	PD-L1− (n = 16)	PD-L1+ (n = 33)	p

Tumor (n) (%)									
<2.5 cm	9 (32.1)	6 (28.6)	0.788	7 (66.7)	8 (28.6)	0.720	4 (25.0)	11 (33.3)	0.743
>2.5 cm	19 (67.9)	15 (71.4)		14 (66.7)	20 (71.4)		12 (75.0)	22 (66.7)	

Grade (n) (%)									
2	9 (32.1)	1 (4.8)	**0.030**	8 (38.1)	2 (7.1)	**0.012**	6 (40.0)	4 (11.8)	**0.024**
3	19 (67.9)	20 (95.2)		13 (61.9)	26 (92.9)		9 (60.0)	30 (88.2)	

ALI (n) (%)									
Negative	12 (42.9)	7 (33.3)	0.498	10 (47.6)	9 (32.1)	0.271	5 (31.3)	14 (42.4)	0.452
Positive	16 (57.1)	14 (66.7)		11 (52.4)	19 (67.9)		11 (68.8)	19 (57.6)	

Ki-67 score (n) (%)									
<50	14 (48.3)	5 (25.0)	0.100	12 (54.5)	7 (25.9)	**0.041**	9 (60.0)	10 (29.4)	**0.043**
≥50	15 (51.7)	15 (75.0)		10 (45.5)	20 (74.1)		6 (40.0)	24 (70.6)	

pN (n) (%)									
0	14 (50.0)	7 (33.3)	0.243	10 (47.6)	11 (39.3)	0.560	4 (25.0)	17 (51.5)	0.079
≥1	14 (50.0)	14 (66.7)		11 (52.4)	17 (60.7)		12 (75.0)	16 (48.5)	

pT (n) (%)									
T1–T2	22 (62.9)	6 (42.9)	0.201	16 (45.7)	5 (35.7)	0.523	10 (62.5)	25 (75.8)	0.501
T3–T4	13 (37.1)	8 (57.1)		19 (54.3)	9 (64.3)		6 (37.5)	8 (24.2)	

Stage (n) (%)									
1	5 (23.8)	3 (10.7)	0.467	6 (21.4)	2 (9.5)	0.295	3 (18.8)	5 (15.2)	**0.049**
2	8 (38.1)	13 (46.4)		13 (46.4)	8 (38.1)		3 (18.8)	18 (54.5)	
3	8 (38.1)	12( 42.9)		9 (32.1)	11 (52.4)		10 (62.5)	10 (30.3)	

Recurrence (n) (%)									
Present	7 (25.0)	6 (28.6)	0.779	5 (23.8)	8(28.6)	0.709	6 (37.5)	7 (21.2)	0.304
Absent	21 (75.0)	15 (71.4)		16 (76.2)	20(71.4)		10 (62.5)	26 (78.8)	

ALI: angiolymphatic invasion.

**Table 2:** Association of LAG-3 iTILs, LAG-3 sTILs, PD-L1 IC, and PD-L1 TC.

	LAG-3 sTIL		p-value	LAG-3 iTIL		p-value
	Negative	Positive		Negative	Positive	
PD-L1(IC) n (%)			**0.008**			**0.049**
Negative	11(50.0)	4 (14.8)		12 (41.4)	3 (15.0)	
Positive	11(50.0)	23(85.2)		17 (58.6)	17 (85.0)	
PD-L1(TC) n (%)			0.112	28 (96.6)	14 (70.0)	**0.014**
Negative	21(95.5)	21(77.8)		1 (3.4)	6 (30.0)	
Positive	1(4.5)	6(22.2)				

IC: Immune cell, TC: tumor cell.

**Table 3:** Spearman correlations of LAG-3 sTIL, PD-L1 IC, PD-L1 TC, and LAG-3 iTIL.

		LAG-3 sTIL	PD-L1 IC	PD-L1 TC	LAG-3 iTIL
LAG-3 sTIL	r		**0.380** ^**^	0.251	**0.750** ^**^
	p-value		0.007	0.082	0.000
	n		49	49	49
PD-L1 IC	r	**0.380** ^**^		0.271	0.281
	p-value	0.007		0.059	0.050
	n	49		49	49
PD-L1 TC	r	0.251	0.271		**0.373** ^**^
	p-value	0.082	0.059		0.008
	n	49	49		49
LAG-3 iTIL	r	**0.750** ^**^	0.281	**0.373** ^**^	
	p-value	0.000	0.050	0.008	
	n	49	49	49	

p < 0.05, r: Spearman correlations.
